# A comparative genomics approach revealed evolutionary dynamics of microsatellite imperfection and conservation in genus *Gossypium*

**DOI:** 10.1186/s41065-017-0034-4

**Published:** 2017-05-18

**Authors:** Muhammad Mahmood Ahmed, Chao Shen, Anam Qadir Khan, Muhammad Atif Wahid, Muhammad Shaban, Zhongxu Lin

**Affiliations:** National Key Laboratory of Crop Genetic Improvement, College of Plant Science & Technology, Huazhong Agricultural University, Wuhan, Hubei 430070 China

**Keywords:** *Gossypium*, Microsatellites, Motif imperfection, Comparative genomics, Evolution

## Abstract

**Background:**

Ongoing molecular processes in a cell could target microsatellites, a kind of repetitive DNA, owing to length variations and motif imperfection. Mutational mechanisms underlying such kind of genetic variations have been extensively investigated in diverse organisms. However, obscure impact of ploidization, an evolutionary process of genome content duplication prevails mostly in plants, on non-coding DNA is poorly understood.

**Results:**

Genome sequences of diversely originated plant species were examined for genome-wide motif imperfection pattern, and various analytical tools were employed to canvass characteristic relationships among repeat density, imperfection and length of microsatellites. Moreover, comparative genomics approach aided in exploration of microsatellites conservation footprints in *Gossypium* evolution. Based on our results, motif imperfection in repeat length was found intricately related to genomic abundance of imperfect microsatellites among 13 genomes. Microsatellite decay estimation depicted slower decay of long motif repeats which led to predominant abundance of 5-nt repeat motif in *Gossypium* species. Short motif repeats exhibited rapid decay through the evolution of *Gossypium* lineage ensuing drastic decrease of 2-nt repeats, of which, “AT” motif type dilapidated in cultivated tetraploids of cotton.

**Conclusion:**

The outcome could be a directive to explore comparative evolutionary footprints of simple non-coding genetic elements i.e., repeat elements, through the evolution of genus-specific characteristics in cotton genomes.

**Electronic supplementary material:**

The online version of this article (doi:10.1186/s41065-017-0034-4) contains supplementary material, which is available to authorized users.

## Background

Microsatellites are DNA structural elements in which a short sequence pattern (motif) is repeated by various numbers and ubiquitously presented in genomes of eukaryotes and prokaryotes. Various mechanisms like replication slippage, unequal crossover, realignment after disassociation of replicating strands, mispairing and base substitution are causative to variations which ultimately lead to extensive polymorphism in microsatellites [1, 2]. Replication slippage events are majorly responsible for extensive repeat length variations, and mispairing of replication strands is key determinant for base substitution in DNA sequence [3]. Moreover, stable wobble mispairing and inefficiency of mismatch repair system could incorporate point mutations anywhere in genome sequence [4]. These processes could impinge on microsatellites and generate repeats with occasional mismatch, motif imperfection, in their repeat units.

During the replication process, insertion or deletion in repeat motif units due to reduced strand specificity or slippage errors could generate length variations. Various models comprehend and illustrate the mechanism of replication slippage event [5]. A single unit repeat is inserted or deleted at a time according to stepwise mutation model [6], while repeat unit variations were found associated to a distinct probability distribution under specific assumptions [7]. Similarly, another model proposed generation of microsatellites as 3′ extension of retrotransposons in a way similar to polyadenylation of mRNA [8]; while, idea of probable generation and insertion of microsatellites in 5′ region and inside the mobile elements further extended the model [9]. On contrary, the mechanisms underlying mispairing and interruptions leading to single nucleotide changes are poorly understood [10].

Non-coding DNA elements, including microsatellites, also experience various evolutionary forces and show high mutation rates (10^−2^ – 10^−6^) in response to selection forces as these usually go unnoticed [11]. Thus, higher mutability and hyper variability of microsatellites could be key determinants of a dynamic equilibrium state in which rapid loss or conservation of non-coding DNA elements exist in species among or within a clade [12]. Various studies reported microsatellite conservation over longer time [13], and recently a study reported conservation up to 450 million years ago (MYA) in vertebrates [14]. Similarly, evolutionary forces might regulate the mechanism which drives fate determination processes of non-coding or coding DNA elements. Thus, conservation and/or deterioration of DNA structural elements over a geographic time period determine impact of species-evolving processes like speciation, diversification, domestication and even duplication through polidization in plant genomes.

Non-coding elements constitute a major proportion of DNA in all forms of life and play a crucial role in modulating coding regions of DNA. Occurrence and applicability of microsatellites in coding sequence (CDS) have elucidated their functional importance [15]. Since microsatellites are implied to regulate biological functions [16], mutations in coding microsatellites could be informative and even point mutation might induce deleterious effects. However, our brief understandings about mechanisms underlying motif imperfection, either through base substitutions or point mutation, cause an eminent impediment in this regard.

In this report, genome-wide motif imperfection pattern were discerned in 13 plant genomes of diverse origins. The relationship between repeat length and degree of motif imperfection with its impact on the genomic abundance of imperfect microsatellites was determined, particularly for *Gossypium* species. Moreover, evolutionary patterns of microsatellite conservation and/or loss among *Gossypium* species were also established to ascertain structural consequences of whole genome duplication and allopolypolidization events through evolution of cultivated cotton tetraploids.

## Methods

### Genome assemblies of 13 plant species

The genome sequences of thirteen plant species were investigated comprising ten dicots and three monocots. The dicot species belonged to family Brassicaceae (*Arabidopsis thaliana* and *Brassica rapa*), Fabaceae (*Glycine max* and *Phaseolus vulgaris*), Salicaceae (*Populus trichocarpa*) and majorly to Malvaceae (*Gossypium arboreum*, *G. raimondii*, *G. hirsutum*, *G. barbadence* and *Theobroma cacao*). The three monocots belonged to family Poaceae (*Brachypodium distachyon*, *Oryza sativa* and *Zea mays*). In addition, coding sequences of annotated genes were also searched for imperfect repeats among four *Gossypium* species. The genome assemblies and CDS sequences of *Gossypium* species were retrieved from Cottongen [17], while genome sequences of other nine plant species were obtained from NCBI Genome Portal [18]. The name of each species was abbreviated to four letters where first capital letter denoted to *genus* and trailing three letters as *species* name.

### Imperfect microsatellites identification

The SciRoKo (v3.4) program utility [19] was used with default imperfect search parameters to identify imperfect microsatellites of varying motif length from 1 to 6 nucleotide (nt) that were designated as 1-nt, 2-nt, 3-nt, 4-nt, 5-nt and 6-nt among 13 plant genomes. Imperfect search criteria regarding repeat length and mismatch penalty were modified, as previously described [20]. Maximum number of successive mismatch was set to ‘3’, and minimum repeat length was set to 15 nt. Compound microsatellites were excluded where maximum distance for association between two repeats was less than 100 nt. For motif standardization, each microsatellite motif underwent a two-step complete standardization procedure. In the first step, repeat motifs like “AG” and “GA” were all categorized as “AG” and termed as partially standardized motifs. Subsequently, in the second step, reverse complement sequence of motifs was determined and all microsatellites were assigned to completely standardized motifs. Eventually, motifs like “AG”, “GA”, “TC” and “CT” were all designated to a single group “AG”” for further analysis. Such complete motif standardization resulted in 2, 4, 10, 33, 102 and 350 group categories for 1-nt, 2-nt, 3-nt, 4-nt, 5-nt and 6-nt repeat motifs, respectively.

### Motif imperfection and repeat length analyses

The genomic abundance of imperfect repeats was ascertained with varying mismatch counts and repeat lengths. Initially, short length repeats were selected and motif imperfection was employed to discriminate between perfect (with no mismatch) and imperfect microsatellites (with mismatch >0) among all genomes. It was noticed that loci with repeat length < 20 nt did not carry mismatch, thus were excluded from further analysis. While, repeats <25 nt had only ‘1’ mismatch per locus and there were up to ‘2’ mismatch in repeat loci <30 nt. The two datasets were mined separately, one having genomic abundance of microsatellites with ‘0’ or ‘1’ mismatch (no or low degree imperfection). While, other comprised of exactly ‘1’ or ‘2’ mismatch (low or higher degree imperfection) for repeats of length 20 to 24 nt (dataset I) and 25 to 29 nt (dataset II), respectively.

Thereafter, three different analytical strategies were employed. Firstly, principal coordinates method [21] was used to conduct generalized discriminant analysis on both datasets separately. The analysis generated canonical axes scores which were used to determine correlation among the variables, and statistical significance was estimated by permutation (*n* = 9999) method implemented in ‘CAP’ program [22]. Moreover, the R package [23] was also used to test the significance of canonical correlations by add-on package ‘CCP’ [24]. Secondly, longer microsatellites of length 35, 40, 45, 50, 55, 60, 65, 70, 75 and 80 nt were targeted with different levels of imperfection (1, 2, 3 and 4 mismatch per loci). The relationships between motif imperfection and varied length repeats were estimated using permutational analysis of variance *PERMANOVA* [25] by calling “adonis” function under package vegan [26], and statistical significance of *F-statistic* was measured (0.05) after permutations (*n* = 999) in R environment. Thirdly, the genomic abundance of imperfect microsatellites was related to mismatch counts and repeat length through *t-statistic* and significant difference were determined (0.05). A linear regression model was fitted to mismatch count and repeat length in each *Gossypium* species.

### Transposable elements (TEs) distribution of perfect vs imperfect microsatellites

Perfect (no mismatch) and imperfect repeats (mismatch ≥1) were compared for relatedness to nearby intact TEs. Each chromosome was considered as independent segment, and TEs abundance was determined among the four cotton genomes in vicinity of microsatellites (500 nt flanked region on both sides) for both sets of repeats. Then, a linear regression model was fitted to the repeat density and TEs abundance in each *Gossypium* species.

### Motif imperfection in coding region of *Gossypium* species

Annotated coding sequences were searched for imperfect repeats under default criteria. All publically available annotated single nucleotide polymorphism (SNP) markers sequences [17] for cotton genomes were downloaded and employed for functional characterization of imperfect microsatellites in coding region.

### Microsatellites conservation among *Gossypium* species

For conservation analysis, microsatellite libraries were developed separately for each species containing repeats flanked with 350 nt on both sides. Because of the drawbacks pertained to sequencing homo-polymers, 1-nt repeats were excluded. Moreover, all compound and overlapping sequences were filtered out and Basic Local Alignment Search Tool (BLAST) was used to validate uniqueness of each locus by NCBI BLAST 2.2.31 [27] through self-BLASTing each library and discarding false positives. A custom perl script was used to hard mask repeat sequences for each locus, and genome to sub-genome (diploid vs sub-diploid in tetraploids) BLAST searches was conducted among libraries. The output was filtered for algorithms like alignment ≥50% of query cover, identity ≥70% and E_value 10^−10^. Since a whole genome duplication (WGD) event was reported through cotton tetraploids’ evolution [28], duplication of each progenitor loci was allowed up to two physical positions for pair wise comparisons (A_2_ vs A_T_ & D_5_ vs D_T_) among homologous chromosomes. The results were validated in reciprocal BLAST searches. Thereafter, conserved microsatellites proportion was employed to develop an intuitive diagram for genome-wide microsatellite conservation by motif size using circos tool [29].

### Estimate of microsatellite decay during paleopolyploidization

The WGD event involved through paleopolyploidization of cultivated tetraploid species was estimated with varied divergence time [30]. A representative median divergence time as ~6 MYA was used. Assuming steady loss of microsatellite loci over the period of divergence time, an exponential fitted decay function was employed to estimate “comparative exponential decay” of microsatellite in *G. hirsutum* and *G*. *barbadence*. On contrary, the “relative decay” estimates of varying motif repeats in tetraploids were determined by comparing proportion of conserved repeats. Thereafter, “relative decay” and “comparative exponential decay” were fitted to an exponential decay function. All non-parametric test statistics were measured and tested for significance (0.05) in R environment.

### Microsatellite relative abundance

The repeat density patterns in cotton diploids exploited evolutionary footprints regarding impact of paleopolyploidization event on it in three different ways. Firstly, proportional abundance of microsatellites in diploid and tetraploid *Gossypium* species was estimated by comparing their relative abundance in *T. cacao*, a close relative belong to Malvaceae. Secondly, the relative abundance of microsatellites according to motif kind and size in four cotton genomes was determined individually. And lastly, comparative abundance of standardized 2-nt motif was analyzed in four cotton genomes. All non-parametric test statistics were measured and tested for significance (0.05) in R environment.

## Results

### Frequency and distribution of microsatellites among monocots and dicots

Microsatellite distribution of varying motif length (1–6 nt) was examined in ten dicots and three monocots. Generally, 2-nt and 3-nt repeats were found in higher percentage in dicots and monocots (Table 1), respectively. While, Atha exhibited almost equal proportion of three motifs (1–3 nt). A plentiful abundance of 3-nt microsatellite was observed in Osat (33.67%) and the trend persisted among Bdis and Zmay. Among dicots, the proportion of 2-nt repeats prevailed and Brap featured the highest proportion of it (39.67%). However, frequency of 5-nt microsatellites predominated and featured specific to four *Gossypium* genomes. Reckoning to the four cotton genomes, a drastic reduction in percentage of 2-nt repeats was observed in tetraploids (Ghir and Gbar) up to 11.95%. While, density of microsatellites with short motif repeats decreased, those with longer motifs (4–6 nt) exhibited incessant abundance in tetraploids compared to their progenitors (Table 1).

**Table 1:** Microsatellite distribution as percent motif abundance (%) among 13 plant genomes

Species	Abbreviation	Mono- (1-nt)	Di- 2-nt)	Tri- (3-nt)	Tetra- (4-nt)	Penta- (5-nt)	Hexa- (6-nt)
*A. thaliana*	Atha	24.05	22.48	24.48	7.36	15.29	6.33
*B. rapa*	Brap	8.77	39.68	18.44	10.12	16.08	6.91
*G. arboreum*	Garb	10.02	22.02	14.90	14.12	28.93	10.01
*G*. *barbadence*	Gbar	6.43	11.95	17.12	18.74	33.05	12.72
*G. hirsutum*	Ghir	3.72	11.95	17.58	19.31	34.37	13.07
*G*. *raimondii*	Grai	6.89	19.78	14.78	17.85	29.88	10.82
*P. trichocarpa*	Ptri	14.82	27.05	19.22	12.53	16.84	8.54
*G. max*	Gmax	12.58	32.54	17.88	11.62	18.60	7.79
*P. vulgaris*	Pvul	3.94	30.60	20.90	14.00	20.35	10.20
*T. cacao*	Tcac	12.95	32.60	16.92	12.54	19.23	5.76
*O. sativa*	Osat	5.02	21.03	33.67	13.02	17.12	10.15
*B. distachyon*	Bdis	9.49	11.99	30.25	18.10	20.94	11.51
*Z. mays*	Zmay	6.16	19.55	28.39	13.45	20.66	9.51

Our data depicted differential variations in repeat density (per Mb) and frequencies of imperfect microsatellites related to genome sizes among 13 plant genomes (Additional file 1: Table S1). Density of imperfect microsatellites varied from 15.61 to 49.07%, and an eminent variation pattern was observed in monocots compared to dicots. Lower repeat density was noticed for species with genome sizes bigger than 1 Gb (10^9^ bases) like Zmay, Ghir, Gbar and Garb and the trend persisted except for Bdis. All species depicted differential variations in imperfection percentage, and species belonging to same genus (*Gossypium*) exhibited varied extent of imperfection. Moreover, differential variations in frequency of imperfect microsatellites was observed among chromosomes of 13 genomes (Additional file 2: Table S2). The four *Gossypium* species contained substantial variations among cognate homologous chromosomes.

### Motif imperfection and repeat length relationship

**Fig. 1:**
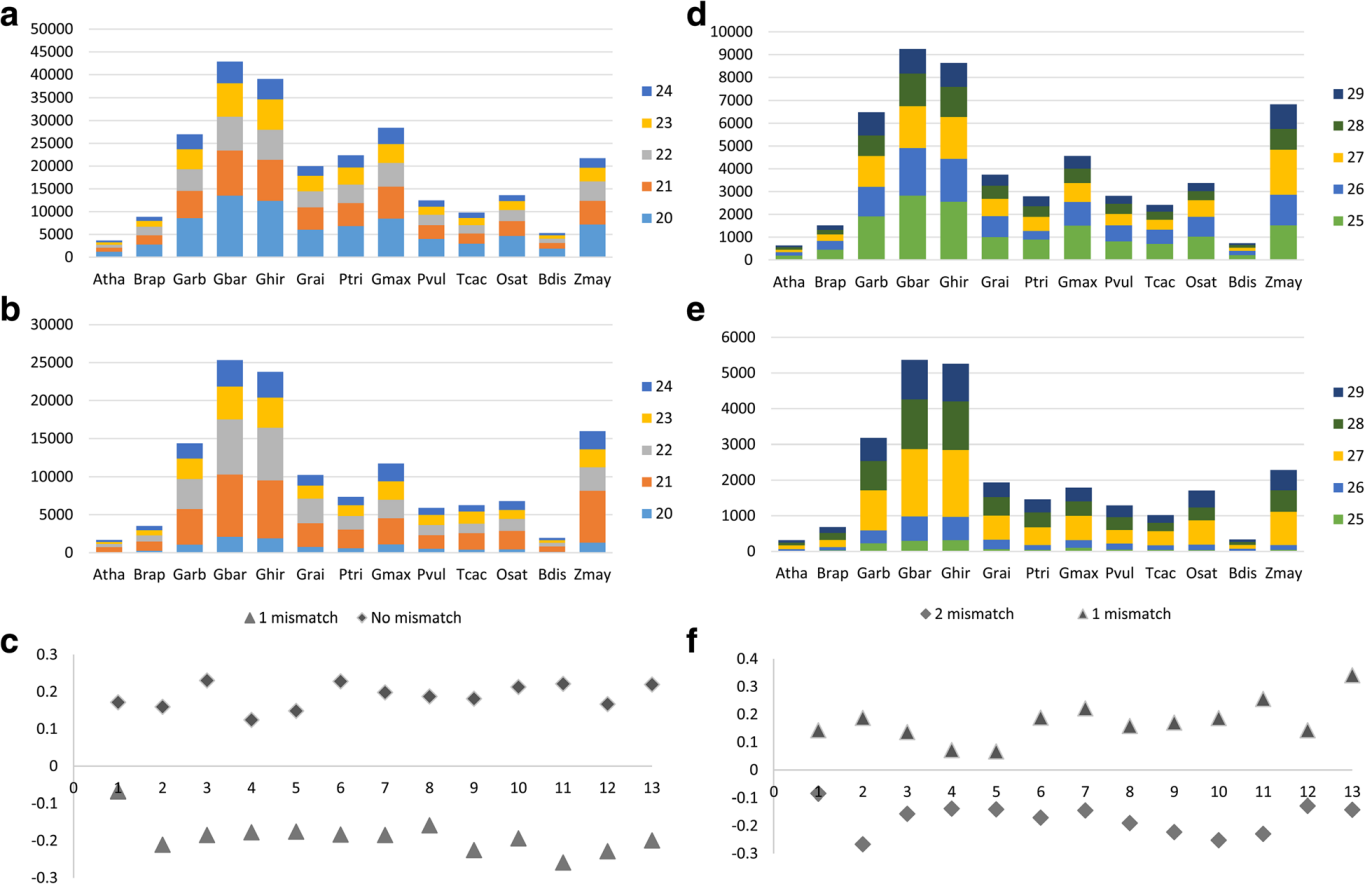
Frequency variations of various mismatch repeats among 10 dicot [*A. thaliana* (Atha), *B. rapa* (Brap), *G. arboreum* (Garb), *G*. *barbadence* (Gbar), *G. hirsutum* (Ghir), *P. trichocarpa* (Ptri), *G. max* (Gmax), *P. vulgaris* (Pvul) and *T. cacao* (Tcac)] and 3 monocots [(*O. sativa* (Osat), *B. distachyon* (Bdis) and *Z. mays* (Zmay)] shown as stacked bar graphs. Frequency variations of length repeats (20, 21, 22, 23 and 24 nt) harboring (**a**) no mismatch or (**b**) exactly ‘1’ mismatch were presented and (**c**) canonical axes plotted as scatter plot. While frequency variations of length repeats (25, 26, 27, 28 and 29 nt) which contained (**d**) exactly ‘1’ mismatch or (**e**) exactly ‘2’ mismatch presented and (**f**) canonical axes plotted as scatter plot

The average length of imperfect microsatellites increased over perfect ones among 13 plant species (Additional file 3: Table S3). Based on significant canonical variations observed for both datasets (dataset I and II), the genomic abundance of microsatellites was found related to variations in repeat length and motif imperfection. The results showed a significant relationship between perfect and ‘1’ mismatch repeats where first squared canonical correlation to be 0.958 (*p* = 0.0002). Likewise, a significant canonical correlation was determined with first squared canonical variation to be 0.926 (*p* = 0.0008) for dataset II in which ‘1’ and ‘2’ mismatch repeats of varied lengths (25, 26, 27, 28, and 29 nt) were included. In dataset I, ‘1’ mismatch repeats explained lesser canonical variation while ‘2’ mismatch repeats commuted in dataset II (Fig. 1). These findings affirmed that repeat length and motif imperfection were somehow modulated in subordinated ways.

Degree of motif imperfection (mismatch count) could be another factor whose relationship with repeat length might determine genomic abundance of imperfect microsatellites. Outcome of *PERMANOVA* analysis suggested a significant relationship among mismatch count, repeat length and frequency of imperfect microsatellites. The variation in genomic abundance of imperfect microsatellites was significantly related to repeat length (*F* = 8.050, *p* = 0.0029) and motif imperfection (*F* = 1.495, *p* = 0.0055). These findings speculated existence of an evolutionary mechanism which determined length and motif heterogeneity of microsatellites and the pattern prevailed among the studied set of genomes.

Pair-wise *Pearson* correlation estimates were calculated by controlling motif imperfection (Additional file 4: Table S4a-b) for different repeat lengths (35, 40, 45, 50, 55, 60, 65, 70 and 75 nt). The results established strong relationships among different length microsatellites carrying varying mismatch (Table 2). Genomic abundance of ‘1’ mismatch repeats was found more related to ‘2’ mismatch repeats, and the relationship maintained with ‘3’ mismatch repeats. The scenario became more stringent for pair-wise comparisons between ‘2’ mismatch and ‘3’ mismatch repeats. Thus, the genomic abundance of loci with lower degree of imperfection (1 mismatch) was related to varying repeat lengths. Moreover, repeats with low imperfection (1 mismatch) seemed to modulate genomic abundance of repeats with moderate imperfection (2 mismatch). Similarly, repeats with higher imperfection (3 mismatch) were more inflected by repeats which entertained ‘2’ mismatch. These findings suggested a significant role of motif imperfection in determining length of repeats.

**Table 2:** Pair-wise correlation (*Pearson*) estimates for imperfect microsatellites

Repeat length (nt)	Mismatch 1 vs mismatch 2	Mismatch 1 vs mismatch 3	Mismatch 2 vs mismatch 3
30 nt	0.983	0.921	0.952
35 nt	0.919	0.919	0.971
40 nt	0.911	0.807	0.894
45 nt	0.493	0.640	0.763
50 nt	0.767	0.545	0.821
55 nt	0.695	0.699	0.822
60 nt	0.558	0.605	0.643
65 nt	0.868	0.742	0.774
70 nt	0.776	0.629	0.423
75 nt	0.896	0.415	0.492
80 nt	0.742	0.852	0.867

The repeat length presented different lengths while frequencies of repeats harbored 1 mismatch (low imperfection), 2 mismatch (moderate imperfection) and 3 mismatch (high imperfection) were compared among 13 plant species. All estimates were significant while underlined were non-significant at 0.05

### Motif imperfection in *Gossypium* genomes

**Fig. 2:**
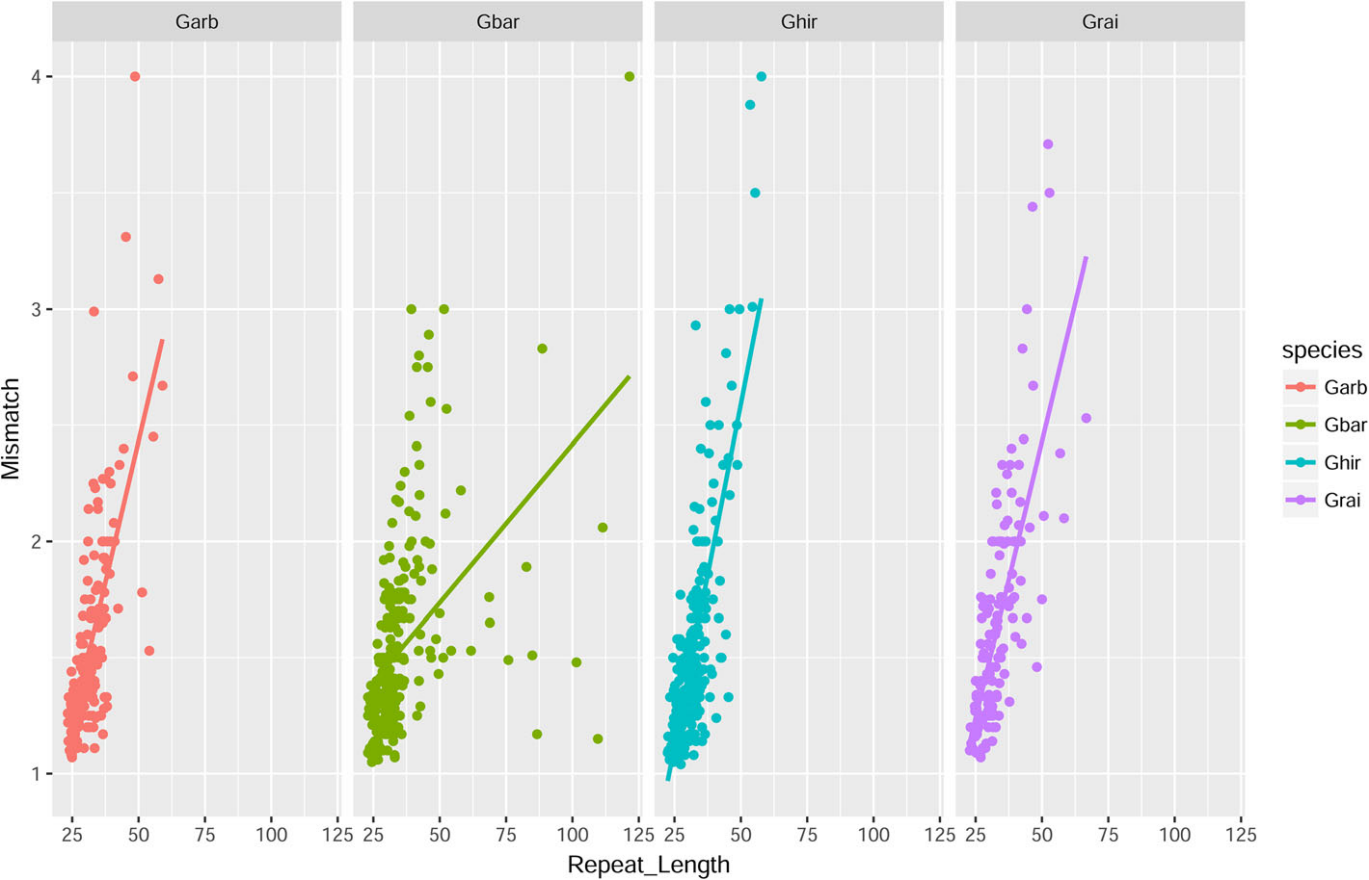
The mismatch (count) and repeat length relationship split among *Gossypium* species. Average mismatch of each standardized motif was compared to their average repeat length (nt) in Garb (*G. arboreum*), Grai (*G*. *raimondii*), Ghir (*G. hirsutum*) and Gbar (*G*. *barbadence*)

To draw deep insights into evolutionary footprints, subsets of mismatched repeats in four *Gossypium* species were considered for following analyses. Linear regression between repeat length and motif imperfection was determined and a prominent relationship was observed in each case (Fig. 2), but with varied degree of variation accounted in the model (Additional file 5: Table S5).

The genomic abundance of intact TEs was determined in 500 nt flanking region (both sides) of perfect and mismatched microsatellites (Additional file 6: Table S6). The two sets of repeats were compared for relatedness between repeat frequency and intact TEs, and significant relationships were observed (Additional file 7: Figure S1a-d). Generally, fewer TEs were observed in Grai and D_T_ sub-genomes; while, higher abundance of TEs discerned for Garb and A_T_ sub-genomes of tetraploids. Moreover, imperfect repeats were more colligated to genomic abundance of TEs than perfect ones except in Garb; where perfect repeats were more related than mismatched ones. This relatedness was further affirmed by *Pearson* correlations and significance determined at 0.05 (Table 3). Correlation values of imperfect repeats were also expectedly higher than perfect ones, but not for Garb in which perfect repeats were most likely imbedded in TEs.

**Table 3:** A comparative correlation estimates between perfect (no mismatch) and imperfect (mismatch ≥1) repeats along with their intact TEs in cotton genomes

Genome/ Sub-genome	Perfect repeats		Imperfect repeats	
	*Pearson* correlation	*p* value	*Pearson* correlation	*p* value
Garb	0.944	1.13E-06	0.743	3.54E-03
Grai	0.631	2.01E-02	0.835	5.23E-04
GhirA_T_	0.788	1.37E-03	0.911	1.47E-05
GhirD_T_	0.615	2.50E-02	0.939	1.39E-06
GbarA_T_	0.869	1.11E-04	0.879	7.49E-05
GbarD_T_	0.648	1.64E-02	0.846	2.58E-04
Ghir	0.833	1.23E-07	0.921	2.63E-11
Gbar	0.876	4.38E-09	0.941	7.22E-13

Garb = *G*. *arboreum*, Grai = *G*. *raimondii*, GhirA_T =_ *G*. *hirsutum* A sub-genome, GhirD_T_ = *G*. *hirsutum* D sub-genome, GbarA_T_ = *G. barbadence* A sub-genome, GbarD_T_ = *G*. *barbadence* D sub-genome, Ghir = *G*. *hirsutum* and Gbar = *G*. *barbadence*

Differential abundance of imperfect repeats along with intact TEs distribution was determined among individual chromosomes. Interestingly, the frequency of imperfect microsatellites and intact TEs for all species was found at par (Additional file 6: Table S6). The higher abundance of intact TEs was observed in Garb than Grai, while vice versa was true for imperfect repeats. Chromosomes A05, A08, A10 and A11 exhibited higher density of both repeat elements, while a mixed pattern was observed for D03, D07 and D12 among tetraploids. Structural anomalies of chromosomes in two genetically distant progenitors, evolutionary processes, biased selection forces, mutations, deletions and translocations of larger DNA segments could be causative to such abrupt differentiations among tetraploids and diploids. Thus, comparative distributive pattern and its relatedness suggested more likely presence of intact TEs wherever a mismatch found in a microsatellite.

### Motif interruptions in coding sequence

Coding sequences of *Gossypium* species were also searched for imperfection and results were summarized in Additional file 8: Table S7. Generally, tri- and hexa- motif repeats prevailed, while only tri- motif repeats accounted for >70% of microsatellites in CDS regions (Table 4). It was expected due to triplet and degenerate nature of codons. The motif imperfection in Grai repeats was elevated and slight increment in average repeat length was noticed. The tandem repeat density was reduced than genomic ones, while the higher proportion of genes in Ghir harbored repeats in exons (Additional file 8: Table S7). Overall, the motif imperfection mechanisms targeted tri- and hexa- repeats in genomic and coding sequences respectively (Additional file 9: Figure S2).

**Table 4:** Summary and characteristics of microsatellites detected in CDS region

Category	Garb^a^	Grai^b^	Ghir^c^	Gbar^d^
Overall SSR density (per Mb)	65.12	61.70	69.48	57.15
3-nt repeat density (per Mb)	44.07	41.43	47.76	38.22
6-nt repeat density (per Mb)	15.95	15.09	17.28	14.99
Average mismatch (Imperfection)	0.25	0.30	0.25	0.22
Average repeat length (nt)	21.15	22.05	21.57	21.19
Genes with tandem repeat in CDS (n)	2471	2846	4964	2992
Genes with mismatched motif (n)	441	475	881	698
Compound microsatellites (n)	71	114	133	202

^a^ *G*. *arboreum* (Garb), ^b^ *G*. *raimondii* (Grai), ^c^ *G*. *hirsutum* (Ghir) and ^d^ *G*. *barbadence* (Gbar)

### Long motif repeats well conserved in *Gossypium* species

**Fig. 3:**
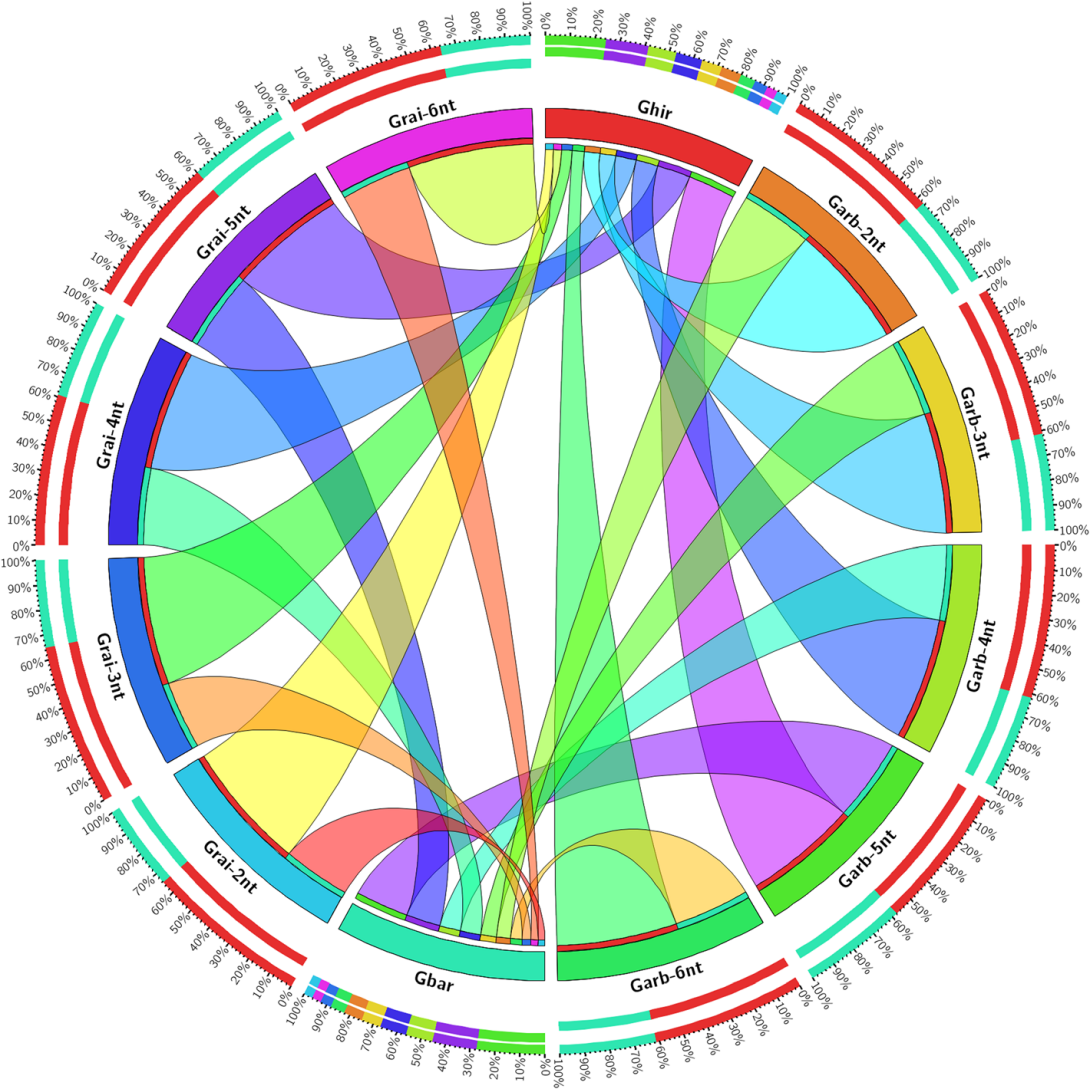
Intuitive diagram shows conserved microsatellite proportion based on motif length. Motif repeats like 2-nt (dinucleotide), 3-nt (trinucleotide), 4-nt (tetra- nucleotide), 5-nt (penta- nucleotide) and 6-nt (hexa- nucleotide) were compared for conservation among Garb (*G. arboreum*), Grai (*G*. *raimondii*), Ghir (*G. hirsutum*) and Gbar (*G*. *barbadence*)

Our data showed predominant abundance of large microsatellites in *Gossypium* species compared to other dicots and monocots. Microsatellite conservation pattern elucidated the possible influence of paleopolyploidization event and traced out the evolutionary footprints in *Gossypium* species. The microsatellites proportion of each A_T_ and D_T_ sub-genome in two cultivated species was ascertained which retained from the progenitors (Fig. 3). The shorter repeats were found less conserved, while repeats with long motifs remained intact as in progenitor species (Additional file 10: Table S8). Among short repeats, the 2-nt microsatellites were found more deteriorated in tetraploids where Gbar retained the lowest proportion from two diploids. On the contrary, 3–6 nt repeats were more conserved, while 5-nt repeats were highly conserved (56.86–78.26%) in both tetraploids. Overall, Ghir retained higher proportion repeats from Garb (66.58%); while, slightly lower proportion retained (61.39%) from another progenitor. Since microsatellites were more deteriorated through series of evolutionary events in Gbar, thus fewer loci retained intact (37.83–43.99%).

### Estimation of microsatellite decay

**Fig. 4:**
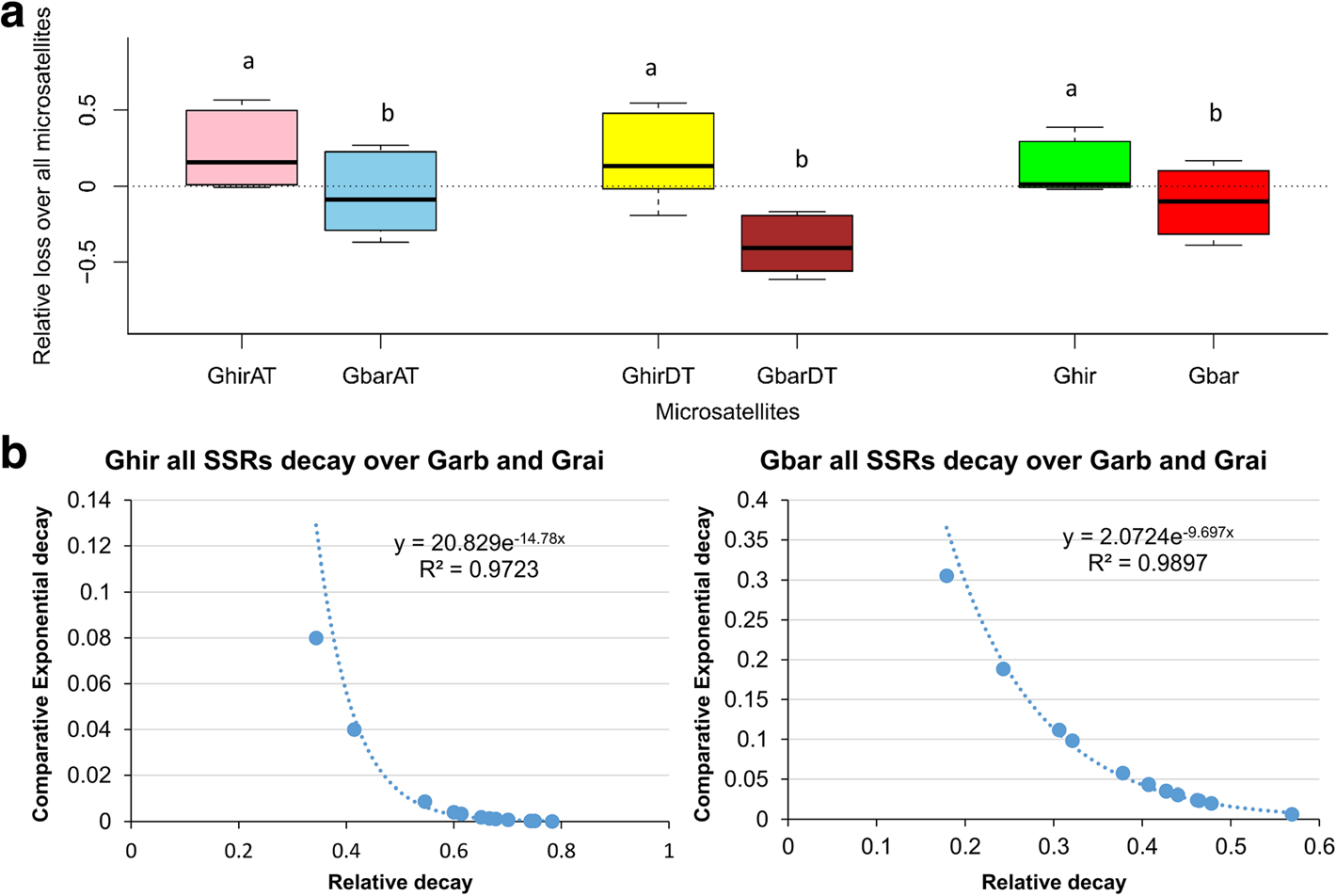
Comprison of microsatellite decay between two cotton tetraploids compared to repeats originated in diploids (Garb and Grai). **a** Relative loss of all SSRs among A_T_ and D_T_ subgenomes of tetraploids (Ghir and Gbar). The *black bar within box* represens median and *dotted horizontal line* represent *y* = 0, while significance was denoted as *lowercase letter*. **b** Comparing relationship of microsatellite relative decay and comparative exponential decay between Ghir and Gbar

The conserved microsatellite proportion and divergence time were fitted to an exponential decay function and microsatellites decay rate of varied length motifs was calculated among *Gossypium* species. A static decay was observed in Ghir for loci from both progenitors except 2-nt repeats; of which D_T_ originated repeats were lost significantly faster (*Friedman statistic* = 48.44, *p* = 0.00001) (Additional file 11: Figure S3a). Whereas in Gbar, 2-nt repeats of D_T_ origin were highly distorted and lost due to significantly faster decay (*Friedman statistic* = 55.083, *p* = 0.00001) (Additional file 11: Figure S3b)*.* In comparison, Ghir retained higher proportion of microsatellites from diploid species than Gbar. Similarly, large motif repeats (3-6 nt) were more evolutionary stable and conserved compared to 2-nt repeats in both A-genome and D-genome of *Gossypium* lineage. Overall, repeats loci in Ghir depicted slower decay of repeats than Gbar (Fig. 4a), while a significant faster decay (*Friedman statistic* = 18.762, *p* = 0.0021) of Grai repeats was observed in tetraploids compared to Garb microsatellites. Considering both “relative decay” and “comparative exponential decay”, all repeat motif exhibited faster decay in Gbar than Ghir (Fig. 4b).

### Biased distribution of dinucleotide repeats in *Gossypium* species

**Fig. 5:**
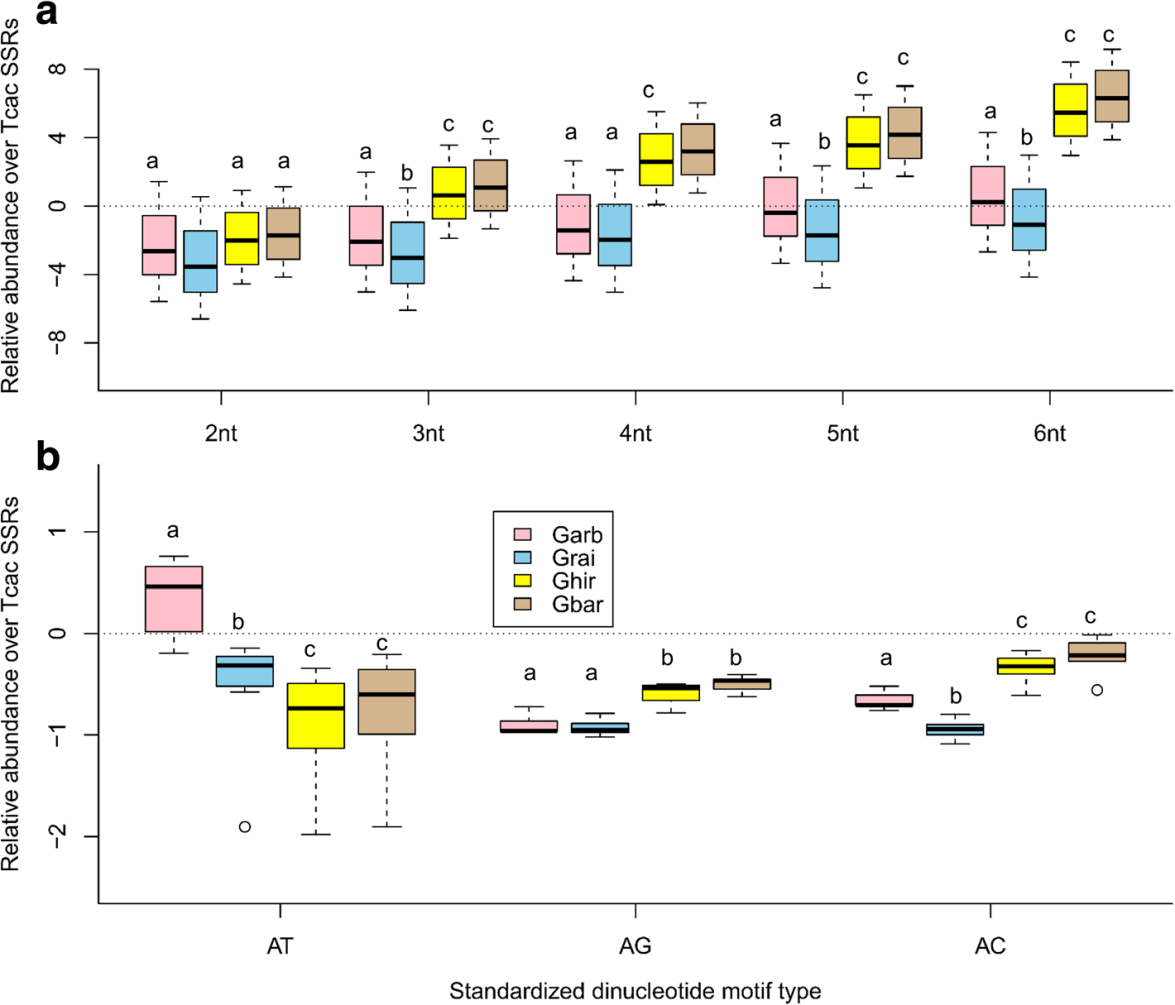
Relative abundances of *Gossypium* microsatellite over *T. cacao* SSRs. Statistical significance was determined using pair-wise *t-test* comparisons and small letters ‘a’, ‘b’ and ‘c’ were assigned to distinct groups significant at 0.05. The *black bar* within box represens median and *dotted horizontal line* represent *y* = 0. **a** Relative abundance of different motif repeats in Garb, Grai, Ghir and Gbar compared to abundance in *T. cacao*. **b** Relative abundance of standardized 2-nt repeat motifs in Garb, Grai, Ghir and Gbar over all 2-nt repeat motifs in *T. cacao*

A WGD event followed by allopolypolidization could be causative to observed evolutionary pattern of microsatellite conservation in *Gossypium* tetraploids. The relative abundance estimates of four *Gossypium* species’ repeats relative to *T. cacao* (as of their closest phylogenetic ancestor) further substantiated the microsatellites decay pattern in *Gossypium* lineage (Additional file 12: Figure S4a-d). The short (1–3 nt) repeats were found less abundant in Garb (*Kruskal-Wallis statistic* = 24.15, *p* = 0.0002) and Grai (*Kruskal-Wallis statistic* = 21.68, *p* = 0.0006), while only 1-nt and 2-nt were drastically lost in Ghir (*Kruskal-Wallis statistic* = 17.23, *p* = 0.0041) and Gbar (*Kruskal-Wallis statistic* = 17.84, *p* = 0.0031). While comparing relative abundance among cotton genomes, the short repeats (1-nt and 2-nt) prevailed less (*Kruskal-Wallis statistic* = 15.70, *p* = 0.0077) than longer (4-6 nt) ones (Fig. 5a). As decay rates were not determined for 1-nt repeats, thereby 2-nt repeat motif was pointed as scarcely abundant. Moreover, all 2-nt standardized motifs were lost in comparative way, while “AT” motif repeats were more rapidly lost in Ghir and Gbar (Fig. 5b). The ploidization could be a major factor for drastic loss of “AT” standardized motif as despite of ploidy increase, tetraploids conserved lower proportion of “AT” motif repeats than diploids.

## Discussion

The study provided a comprehensive account of microsatellite imperfection in 13 plant genomes and explored motif imperfection and repeat length relationships in *Gossypium* lineage. The microsatellite conservation pattern and evolutionary footprints of non-coding repetitive DNA elements were also discerned in closely related species of Malvaceae family.

Genomic abundance of imperfect microsatellites is well studied in simple organisms like viruses [31], mushroom [32], complex animal [33], insect [34], human [35] and even in green plants [36]. However, a clear understanding of imperfection mechanism, its role in genesis, preserving sequence variations in non-coding repeat elements and their potential impact on gene regulation are not well understood. As focus on evolution of microsatellites got reincarnated with the advent of high throughput sequencing technology [37, 38], this study emphasized on role of motif imperfection in microsatellites stability. Plant species were emphasized as microsatellites are being extensively employed in revealing diversity, heterozygosity and exploiting phenotypic variations in plants [39, 40]. Moreover, microsatellites have also been employed to unravel polyploidy in plants [41]. Evolutionary dynamics of microsatellites were revealed in genus *Gossypium*, a best model to investigate ploidy increase impact on microsatellite imperfection and conservation.

Various tools are available for imperfect repeats search, but SciRoKo [19] was preferred since it provides a utility to efficiently digest large genome datasets into 50 Mb chunks. Moreover, assessing efficiency of various tools was beyond the scope of this study. Our results witnessed predomination of 3-nt and 2-nt repeats in monocots and dicots respectively. Many researchers have reported 3-nt repeats’ abundance as characteristics in monocots [42, 43]. Similarly, predominate abundance of 2-nt repeats has been reported in dicots [44, 45], but paramount abundance of other motifs is not out of the scope. The resultant fluctuations might be owed to variations in search parameters. Appropriate parameters were employed for imperfect microsatellites search in all studied plant genomes (Table 1 & Additional file 1: Table S1). Likewise, the distribution and abundance of imperfect repeats varied among the studied species, and it also varied among homologous chromosomes of species (Additional file 2: Table S2). Previous studies supported varied distribution of imperfect repeats among different species of genus *Drosophila* [46]. The trend was not affected by varying quality of 13 genome assemblies as a consistent pattern of motif imperfection was observed.

The genomic abundance of imperfect microsatellites was intricately related to degree of motif imperfection and repeat length variations. Previously, repeat unit variations were found linked to repeat length and mutations [47, 48], while an equilibrium state was proposed to regulate abundance of mismatched repeats [1]. A varying stability of mismatched repeats due to sequence interruptions has been experimentally tested [49]. While, a study determined relationship between mutation and repeat units and explained why longer repeats likely to undergo contractions and short repeats experience expansion in case of slippage mutations [50]. The longer repeats (>40 nt) with no mismatch or lower imperfection (mismatch 1 and/or 2) were more abundant than those with higher degree of imperfection (Additional file 4: Table S4a-b). Moreover, a significant relationship between mismatch count and repeat length existed among *Gossypium* species (Additional file 5: Table S5). Generally, the longer repeats do not remain stable and immediately split to give shorter repeats as these undergo contraction. However, our results predicted longer repeats were more likely to harbor mismatch, but these repeats were less prone to contraction as motif interruptions could impart stabilizing effect to such repeats [51]. Furthermore, a low to moderate positive correlation between microsatellites and TEs was recurrently reported [52, 53] but not between imperfect microsatellites and TEs. Due to sequence interruptions, the longer repeats were found more related to TEs abundance except for Garb, where perfect repeats were more likely to be present in the vicinity of TEs (Table 3). Thus, it can be speculated that microsatellite genesis, prolonged stability, length variations and motif imperfection of mismatched repeats could be better comprehended when the replication slippage errors, polyadenylation of 5′ and 3′ regions of TEs are considered simultaneously.

Recent studies reported that 5-nt repeats were predominated in Garb and Grai [54, 55]. However, another study reported 6-nt repeats prevailed in Ghir [56]; while our results affirmed that 5-nt repeats were consistently frequent in four sequenced *Gossypium* genomes. Although the different search parameters generated variations, our results were in accordance to all previous reports. Previously, phylogenetic and molecular studies have suggested that genus *Gossypium* diverged from close relatives during *Miocene* about 10–15 MYA [57]. The two progenitor diploids; *G. arboreum* and *G*. *raimondii*, underwent a series of rapid speciation and diversification. While allopolyploids accomplished domestication and diversified distribution in mid *Pleistocene* about 1–2 MYA [58]. Proportion of conserved microsatellites strongly suggested rapid loss of shorter repeats (especially 2-nt repeats) and relative abundance analysis evoked preservation of higher repeats in four sequenced cotton species. It was noted that 5-nt repeats were the second more abundant after 2-nt repeats in *T. cacao*. Thus, it could be speculated that through the series of evolutionary landmarks in *Gossypium* evolution; WGD, allopolypolidization and speciation, 2-nt repeats were drastically lost and 5-nt repeats prevailed until *Gossypium* early diploids evolved. The 2-nt repeat density was higher in cotton diploids than tetraploids, since the loss of shorter repeats could be a gradual process (Table 1).

## Conclusion

This study presented a comprehensive view of motif imperfection pattern in 13 genome assemblies representing five taxonomic families in plant kingdom. It determined the pattern of microsatellite motif imperfection and decay in complex higher plants exhibiting polyploidy and laid foundation for further studies; as plenty of plant genome sequences available to question evolutionary theory about origin of life. The results explicated evolution and stability of motif-interrupted repeats, its relationship with repeat length and its impact on their genomic prevalence in monocot and dicots. Moreover, it exploited clues to evolutionary footprints for predominate abundance of 5-nt repeats, which appeared as a characteristic in genus *Gossypium*. Likewise, 2-nt repeats experienced accelerated decay in *Gossypium* tetraploids. When compared with a close relative *T. cacao*, “AT” repeats started being less abundant in geographic time while *Gossypium* lineage was undergoing paleopolyploidization processes through the evolution of today’s domesticated cotton. Characterization of “AT” motif decay hotspots and the impact of motif decay on modulation of gene regulatory elements is mandatory to fully comprehend the basic underlying mechanisms.

## Additional files

Additional file 1: Table S1. Characteristics of imperfect microsatellites investigated among 13 plant genomes. (XLS 25 kb)

Additional file 2: Table S2. Frequency of imperfect microsatellites distributed chromosomes and imperfection (%) in 13 plant genomes. (XLS 39 kb)

Additional file 3: Table S3. Average length comparison between perfect and imperfect repeats. (XLS 33 kb)

Additional file 4: Table S4. **(a)** Frequency of variable length repeats which harbored mismatch (1, 2, 3 and 4) in repeat motifs across 13 plant genomes; **(b)** Probability values for pair-wise length comparisons among imperfect repeats of 13 plant genomes. (XLS 33 kb)

Additional file 5: Table S5. Regression analysis of motif Imperfection (mismatch count) in relation to repeat length among four *Gossypium* species. (XLS 24 kb)

Additional file 6: Table S6. Chromosomal distribution of perfect and imperfect microsatellites in relation with intact (TEs), embedded in 500 nt flanked region on both sides, among four *Gossypium* species. (XLS 28 kb)

Additional file 7: Figure S1. Comparison between perfect (no mismatch) and imperfect repeats (mismatch ≥1) for correlation of microsatellites with intact TEs frequency in (a) *G. arboreum* (Garb), (b) *G. raimondii* (Grai), (c) *G. hirsutum* (Ghir) and (d) *G. barbadence* (Gbar). (DOC 913 kb)

Additional file 8: Table S7. Motif distribution, density and imperfection of microsatellite repeats in coding sequences of four cotton genomes. (XLS 25 kb)

Additional file 9: Figure S2. Comparing motif imperfection pattern between genomic and coding microsatellites of varying motif sizes (2-6 nt) in *G. arboreum* (Garb), *G. raimondii* (Grai), *G. hirsutum* (Ghir) and *G. barbadence* (Gbar). (DOC 51 kb)

Additional file 10: Table S8. Genome to sub-genome comparison of microsatellite conservation analysis. (XLS 28 kb)

Additional file 11: Figure S3. Relative loss of SSRs by motif length in (a) *G. hirsutum* (Ghir) and (b) *G*. *barbadence* (Gbar). The loss of 2-6 nt SSRs compared to the loss of all SSRs (y = 0, denoted by dotted line). Microsatellites of sub-genome A_T_ are shown in gray filling and D_T_ sub-genome shown in white. (DOC 78 kb)

Additional file 12: Figure S4. Relative abundance of microsatellite for *Gossypium* genomes, (a) *G. arboreum* (Garb), (b) *G. raimondii* (Grai), (c) *G. hirsutum* (Ghir), and (d) *G. barbadence* (Gbar), compared to distribution of *T. cacao* SSRs density by motif length (y = 0, denoted by dotted line). (DOC 153 kb)

## Abbreviations

BLAST: Basic local alignment search tool
CDS: Coding sequences
DNA: Deoxyribonucleic acid
Gb: giga (10^9^) bases
Mb: mega (10^6^) bases
mRNA: messenger RNA
MYA: Million years ago
nt: nucleotide
SNP: Single nucleotide polymorphisms
SSR: Simple sequence repeats
TEs: Transposable elements
WGD: Whole genome duplication
