# Supplementary material for: A comparative genomics approach revealed evolutionary dynamics of microsatellite imperfection and conservation in genus Gossypium
Source: Hereditas. 2017 May 18;154:12. doi: 10.1186/s41065-017-0034-4 (PMC5437633; doi:10.1186/s41065-017-0034-4)
Supplement: Supplementary file 7 — Comparison between perfect (no mismatch) and imperfect repeats (mismatch ≥1) for correlation of microsatellites with intact TEs frequency in (a) G. arboreum (Garb), (b) G. raimondii (Grai), (c) G. hirsutum (Ghir) and (d) G. barbadence (Gbar). (DOC 913 kb) [file 41065_2017_34_MOESM7_ESM.doc]

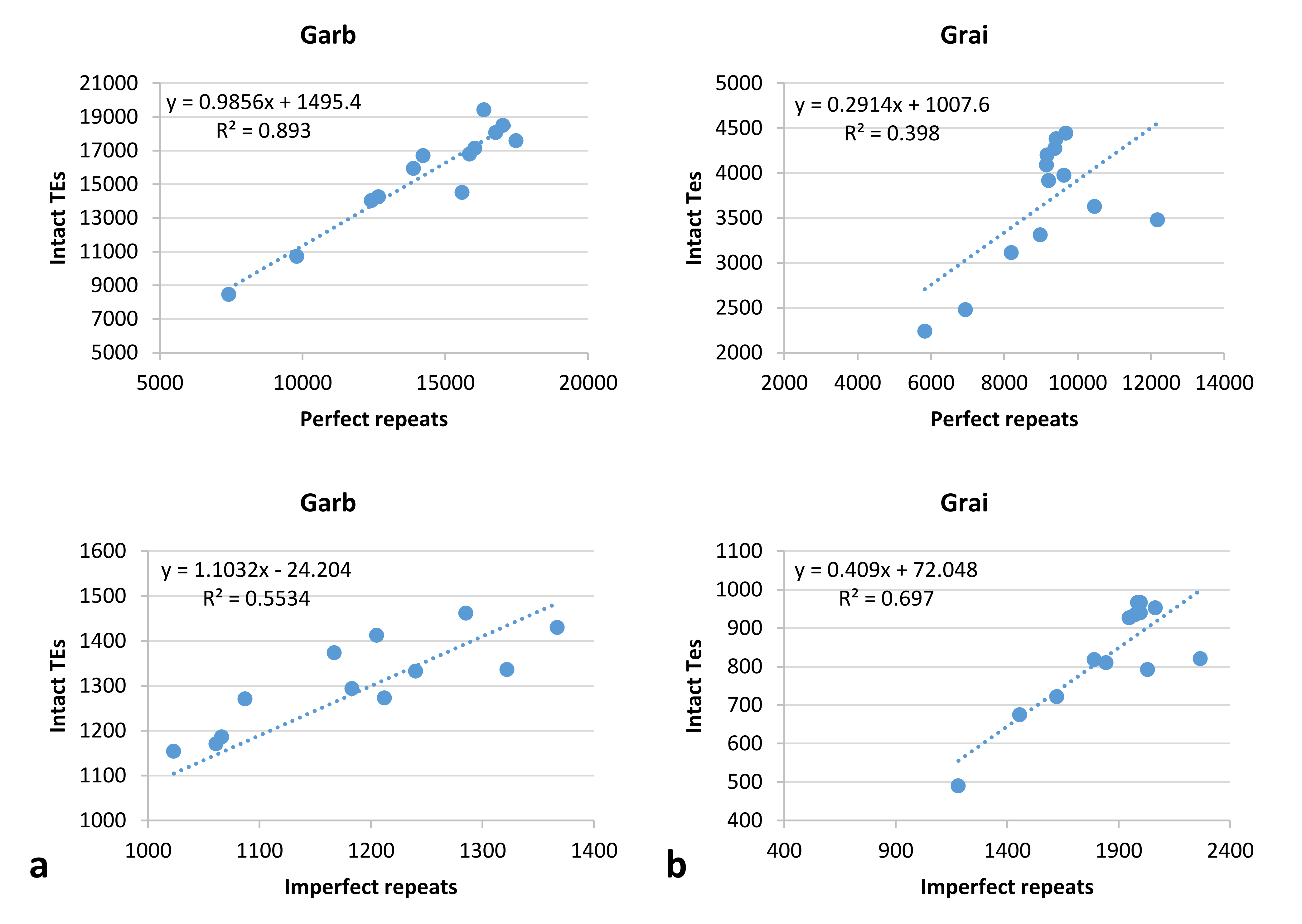


**
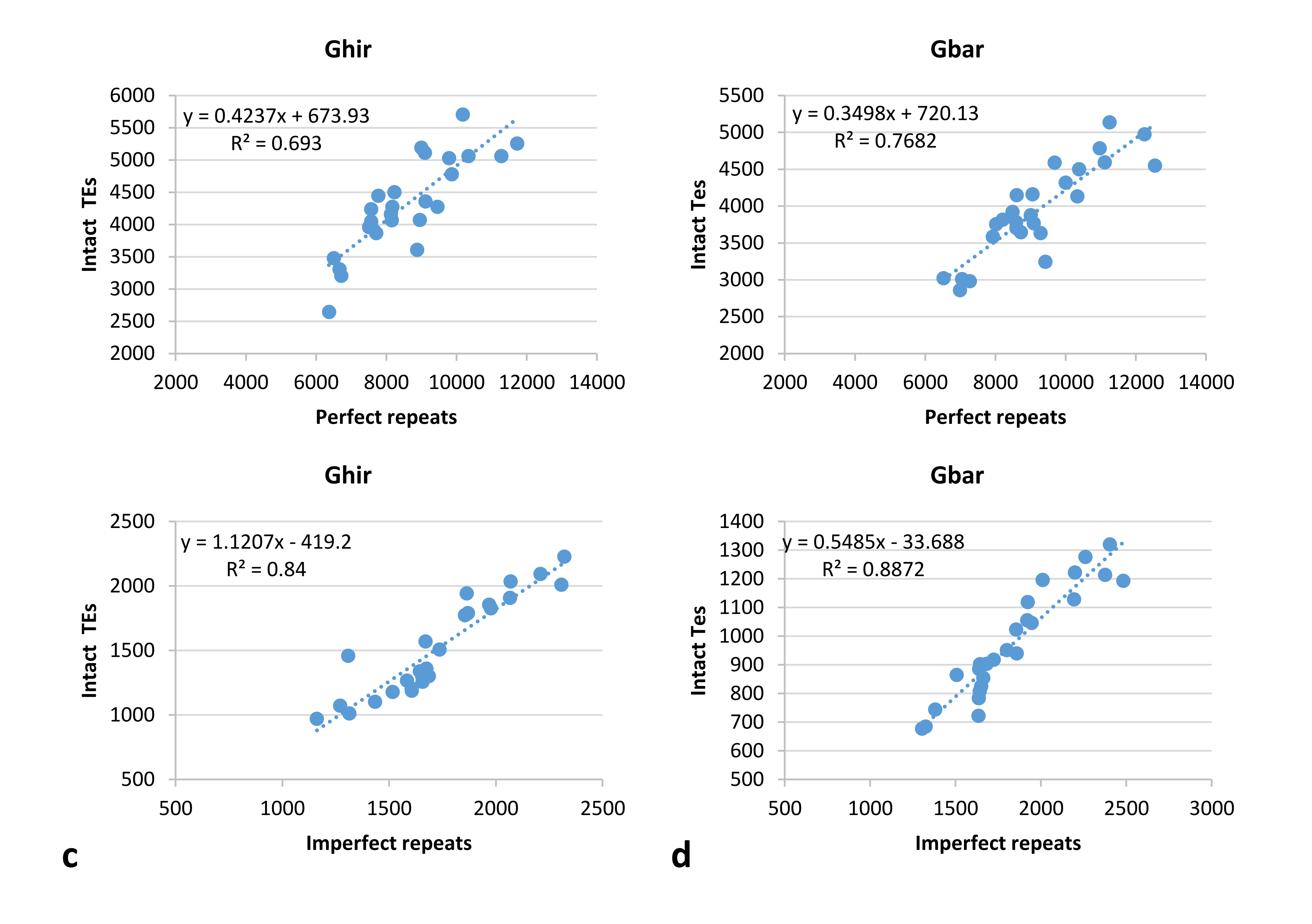
**

**Fig. S1** Comparison between perfect (no mismatch) and imperfect repeats (mismatch > 1) for correlation of microsatellites with intact TEs frequency in (a) *G. arboreum* (Garb), (b) *G. raimondii* (Grai), (c) *G. hirsutum* (Ghir) and (d) *G. barbadence* (Gbar).
