# Supplementary material for: A comparative genomics approach revealed evolutionary dynamics of microsatellite imperfection and conservation in genus Gossypium
Source: Hereditas. 2017 May 18;154:12. doi: 10.1186/s41065-017-0034-4 (PMC5437633; doi:10.1186/s41065-017-0034-4)
Supplement: Supplementary file 9 — Comparing motif imperfection pattern between genomic and coding microsatellites of varying motif sizes (2-6 nt) in G. arboreum (Garb), G. raimondii (Grai), G. hirsutum (Ghir) and G. barbadence (Gbar). (DOC 51 kb) [file 41065_2017_34_MOESM9_ESM.doc]

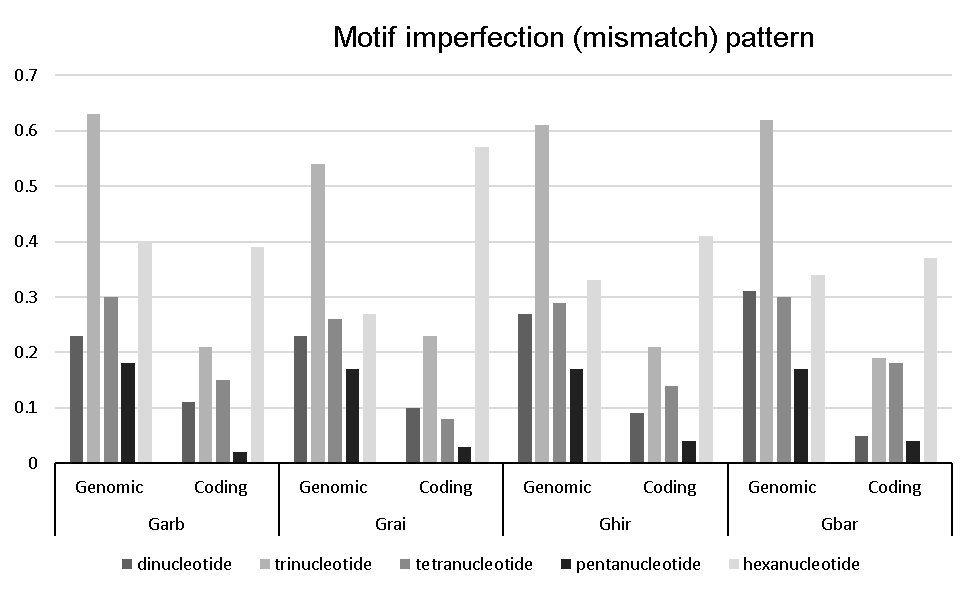


**Fig. S2** Comparing motif imperfection pattern between genomic and coding microsatellites of varying motifs i.e., dinucleotide (2-nt), trinucleotide (3-nt), tetranucleotide (4-nt), pentanucleotide (5-nt) and hexanucleotide (6-nt) in *G.* *arboreum* (Garb), *G.* *raimondii* (Grai), *G.* *hirsutum* (Ghir) and *G.* *barbadence* (Gbar).
