# Supplementary material for: A comparative genomics approach revealed evolutionary dynamics of microsatellite imperfection and conservation in genus Gossypium
Source: Hereditas. 2017 May 18;154:12. doi: 10.1186/s41065-017-0034-4 (PMC5437633; doi:10.1186/s41065-017-0034-4)
Supplement: Supplementary file 11 — Relative loss of SSRs by motif length in (a) G. hirsutum (Ghir) and (b) G. barbadence (Gbar). The loss of 2-6 nt SSRs compared to the loss of all SSRs (y = 0, denoted by dotted line). Microsatellites of sub-genome AT are shown in gray filling and DT sub-genome shown in white. (DOC 78 kb) [file 41065_2017_34_MOESM11_ESM.doc]

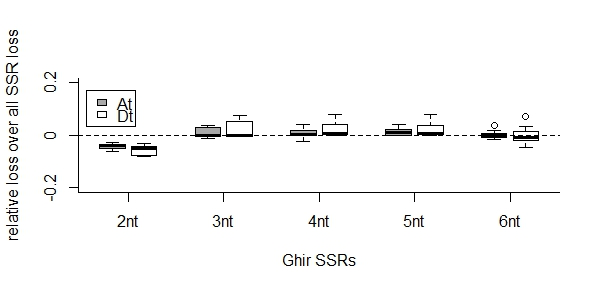
(a)


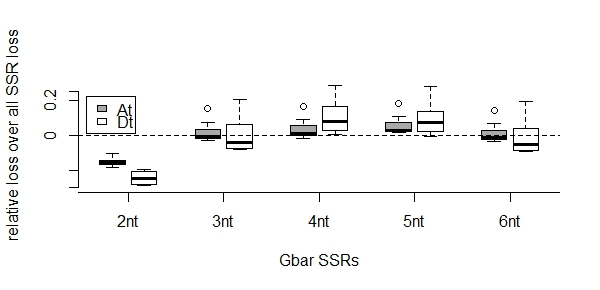
(b)

**Fig. S3** Relative loss of SSRs by motif length in (a) *G*. *hirsutum* (Ghir) and (b) *G*. *barbadence* (Gbar). The loss of 2nt, 3nt, 4nt, 5nt and 6nt SSRs compared to the loss of all SSRs (y=0, denoted by dotted line). Microsatellites of sub-genome A_T_ are shown in gray filling and D_T_ sub-genome shown in white.
