# Supplementary material for: A comparative genomics approach revealed evolutionary dynamics of microsatellite imperfection and conservation in genus Gossypium
Source: Hereditas. 2017 May 18;154:12. doi: 10.1186/s41065-017-0034-4 (PMC5437633; doi:10.1186/s41065-017-0034-4)
Supplement: Supplementary file 12 — Relative abundance of microsatellite for Gossypium genomes, (a) G. arboreum (Garb), (b) G. raimondii (Grai), (c) G. hirsutum (Ghir), and (d) G. barbadence (Gbar), compared to distribution of T. cacao SSRs density by motif length (y = 0, denoted by dotted line). (DOC 153 kb) [file 41065_2017_34_MOESM12_ESM.doc]

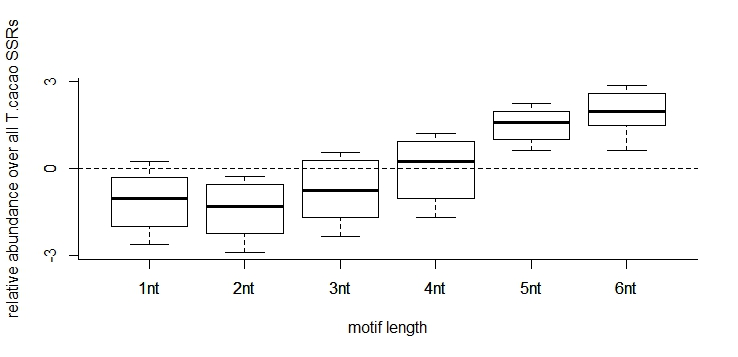
(a)


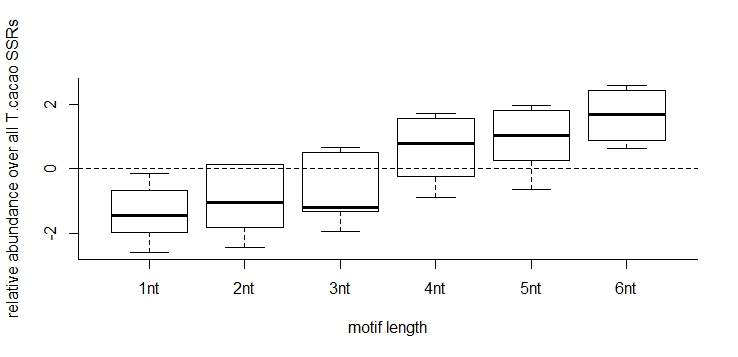
(b)


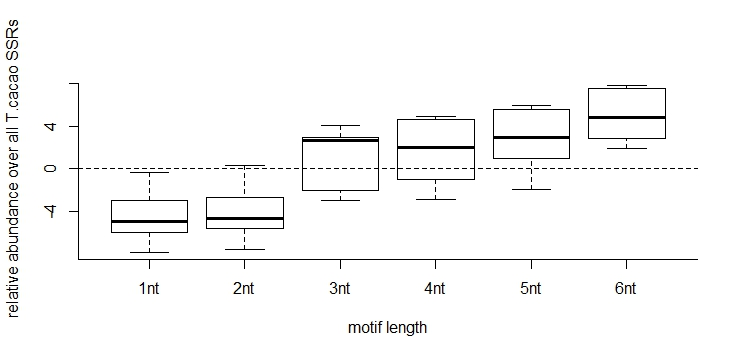
(c)


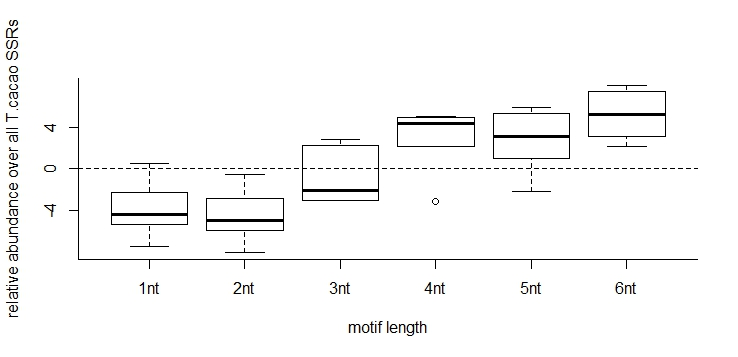
(d)

**Fig. S4** Relative abundance of microsatellite for *Gossypium* genomes, (a) *G.* *arboreum* (Garb), (b) *G.* *raimondii* (Grai), (c) *G.* *hirsutum* (Ghir), and (d) *G.* *barbadence* (Gbar), compared to distribution of *T. cacao* SSRs density by motif length (y=0, denoted by dotted line).
